# Power Doppler ultrasonography findings of eosinophilic fasciitis

**DOI:** 10.1093/rap/rkae067

**Published:** 2024-05-22

**Authors:** Daiki Nakagomi

**Affiliations:** Department of Rheumatology, University of Yamanashi Hospital, Yamanashi, Japan

A 67-year-old woman presented with a 3-month history of fever as well as muscle weakness, swelling, pain and redness in the bilateral forearms (Fig. 1A). On laboratory tests, the eosinophil count was 22/µl, CRP was 10.5 mg/dl and creatine phosphokinase was 3949 IU/l. Contrast-enhanced MRI showed high intensity in the bilateral forearm muscles and fascia. Ultrasonography revealed abnormal power Doppler signals in the bilateral forearm fasciae (Fig. 1B). A biopsy of the fascia revealed eosinophilic infiltration and the patient was diagnosed with eosinophilic fasciitis. The patient was treated with prednisolone (40 mg/day) and her condition is currently in remission. All symptoms and abnormal Doppler signals in the forearms disappeared immediately upon treatment (Fig. 1C and D).

**Figure 1.**
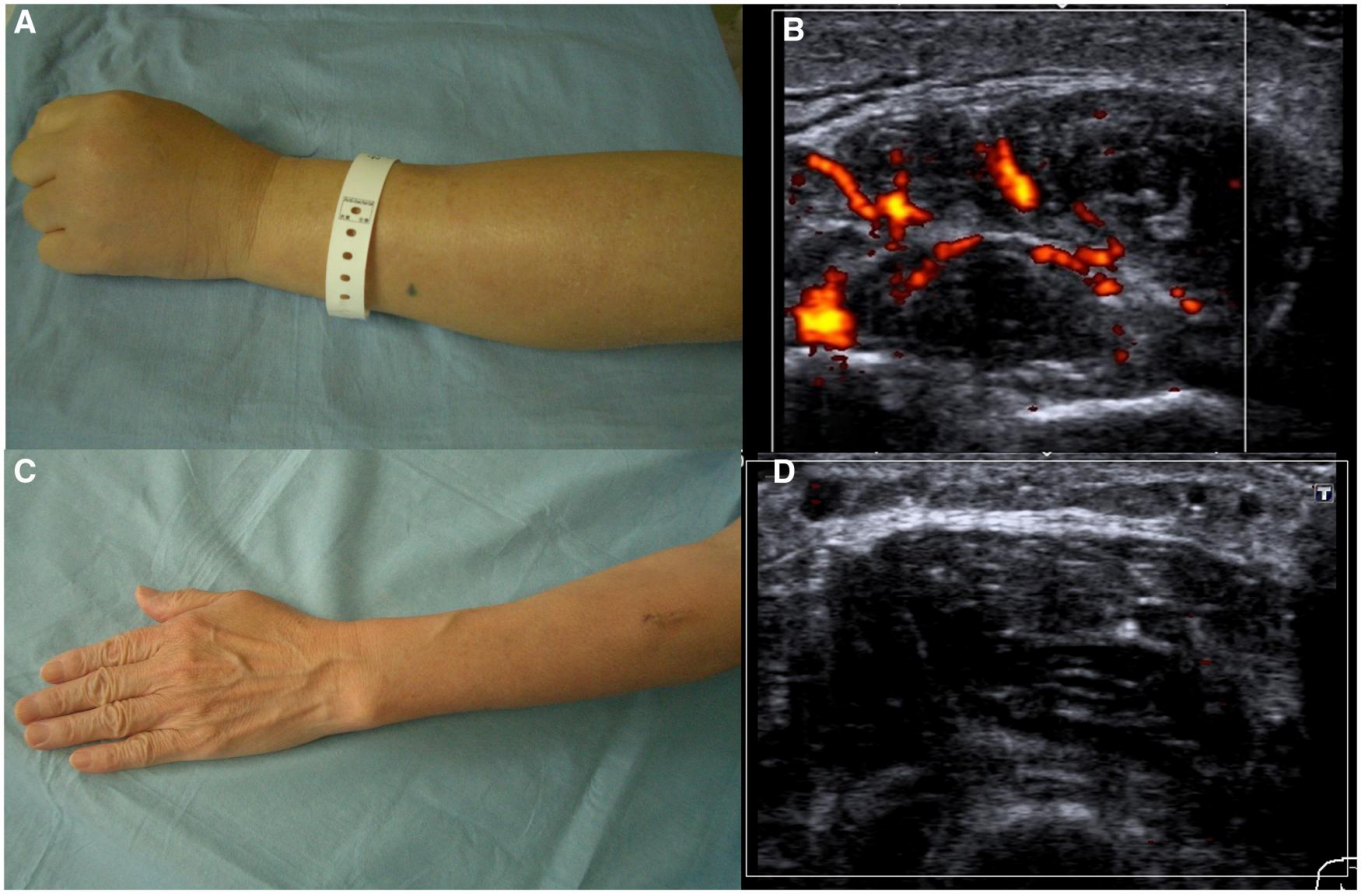
Power Doppler ultrasonography findings of eosinophilic fasciitis. (**A**) Swelling in the left forearm. (**B**) Abnormal Doppler signals in the left forearm fascia. (**C**) No swelling was noted in the left forearm after treatment. (**D**) No abnormal Doppler signals were noted in the left forearm after treatment

Eosinophilic fasciitis is a rare disease characterized by skin thickening and sclerosis and eosinophilia of the blood or fascia. A few studies have demonstrated fascial inflammation using ^18^F-fluoro-2-deoxy-d-glucose PET/CT or MRI [1]. This is the first report to describe the detection of eosinophilic fasciitis as abnormal Doppler signals on ultrasonography. Ultrasonography is a useful imaging tool for the diagnosis of musculoskeletal diseases and can be used to support the diagnosis of eosinophilic fasciitis in the acute phase [2].

## Data Availability

No new data were generated or analysed in support of this research.

## References

[1] Kurimoto R , Ikeda K, Nakagomi D, Nakajima H. Eosinophilic fasciitis illustrated by [^18^F] FDG-PET/CT. Intern Med 2016;55:2321–2. 27523020 10.2169/internalmedicine.55.6937

[2] Au Eong DTM , Cronin O, Biswas A, McKay ND. Ultrasound in the diagnosis and monitoring of eosinophilic fasciitis. Rheumatology (Oxford) 2021;60:e107–8. 33099646 10.1093/rheumatology/keaa498

